# Supplementary material for: Infection with Blastocystis spp. and its association with enteric infections and environmental enteric dysfunction among slum-dwelling malnourished adults in Bangladesh
Source: PLoS Negl Trop Dis. 2021 Aug 18;15(8):e0009684. doi: 10.1371/journal.pntd.0009684 (PMC8405003; doi:10.1371/journal.pntd.0009684)
Supplement: S1 File — (DOC) [file pntd.0009684.s002.doc]

**Infection with *Blastocystis* spp. and its association with enteric infections and environmental enteric dysfunction among slum-dwelling malnourished adults in Bangladesh**

Shah Mohammad Fahim, Md. Amran Gazi, Md. Mehedi Hasan, Md. Ashraful Alam, Subhasish Das, Mustafa Mahfuz, M Masudur Rahman, Rashidul Haque, Shafiqul Alam Sarker, Tahmeed Ahmed


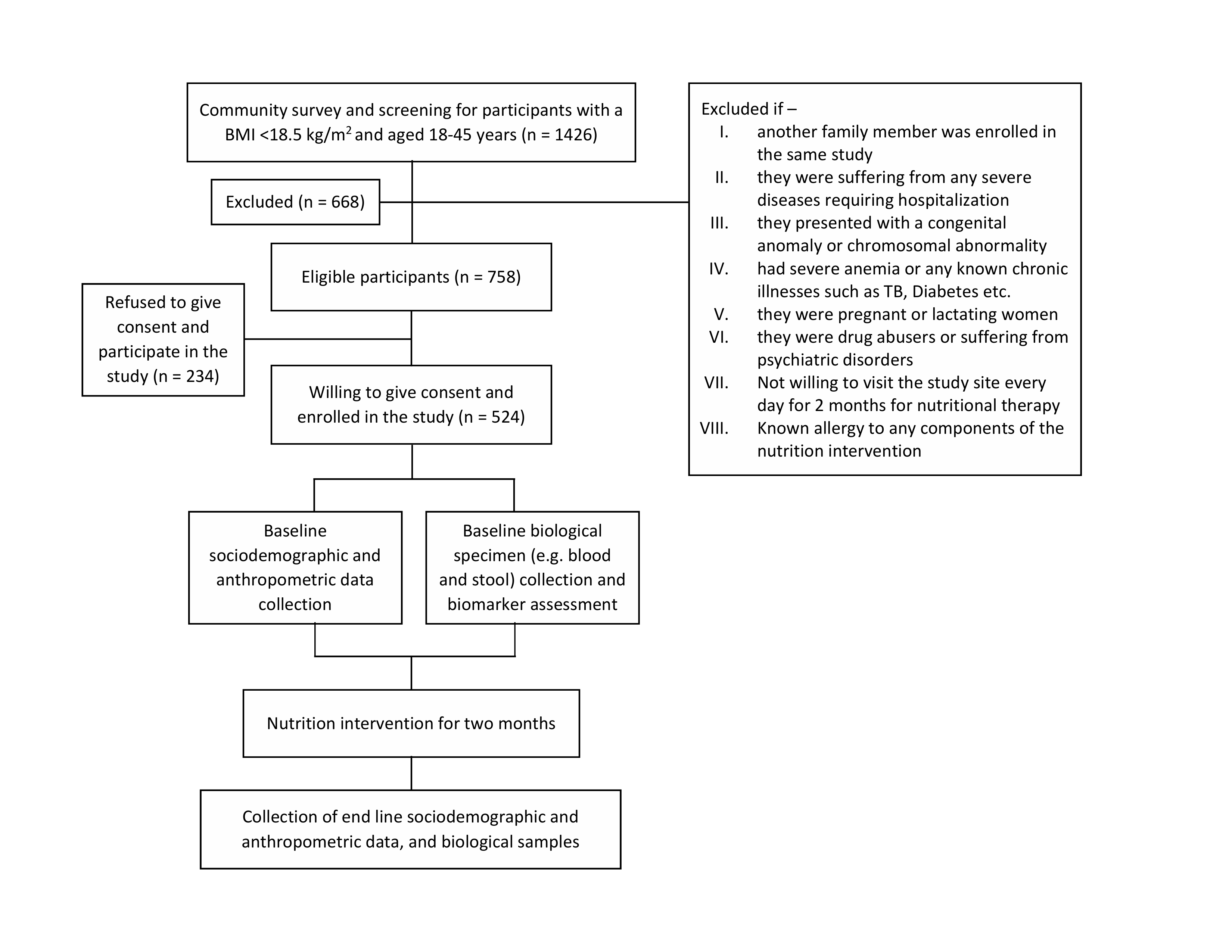


Fig A. Flowchart showing the screening and recruitment of participants in the BEED study. In this analysis, we have only used the baseline data, for instance, sociodemographic information, anthropometric measurements, and results of laboratory analysis done at enrolment.


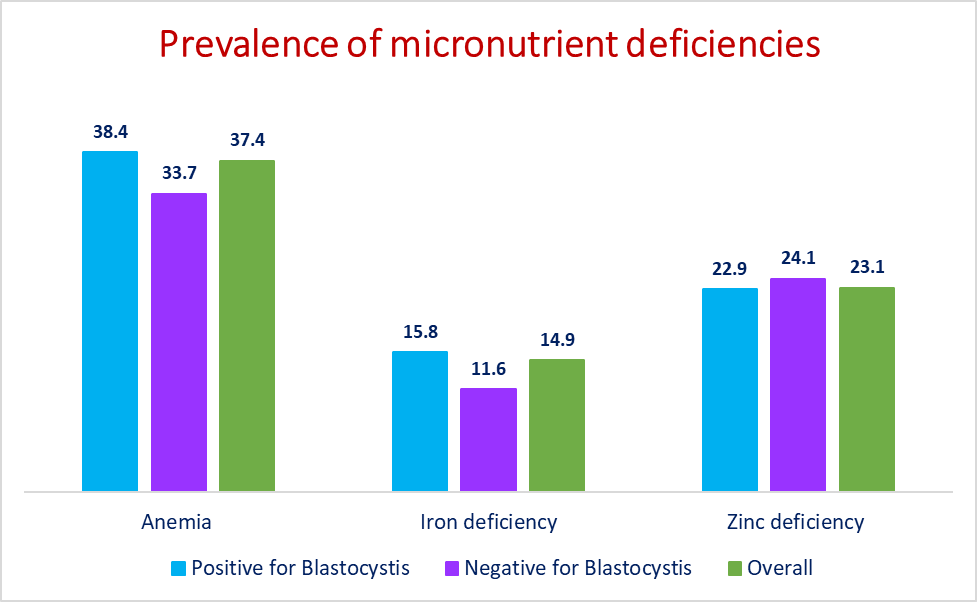


Fig B. Prevalence of micronutrient deficiencies among the study participants (n = 524), overall and in participants with and without *Blastocystis* spp.


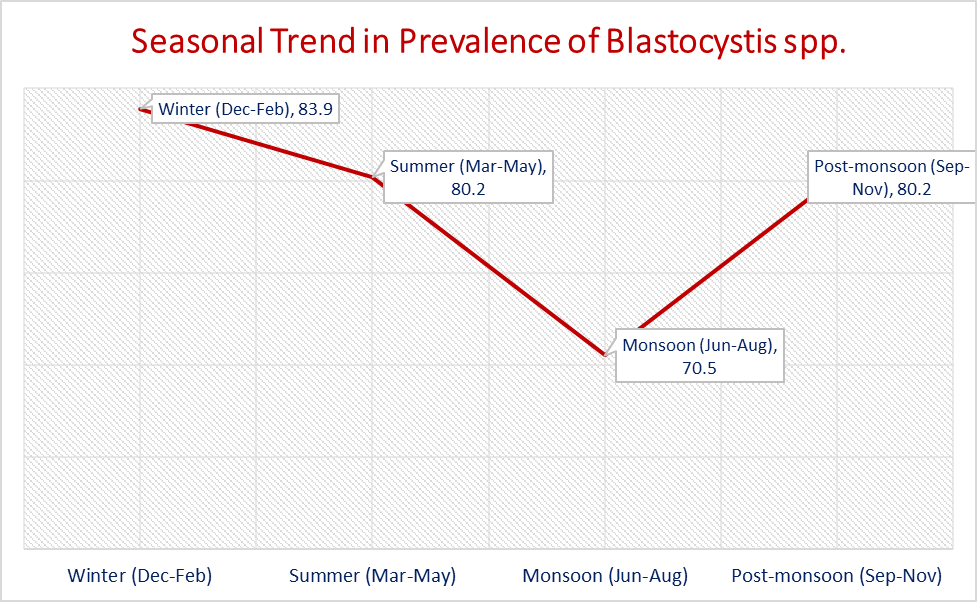
Fig C. Seasonal trend in the prevalence of *Blastocystis* spp. among the study participants (n = 524). The prevalence of *Blastocystis* was greater in winter compared to other seasons, although the difference was statistically insignificant.


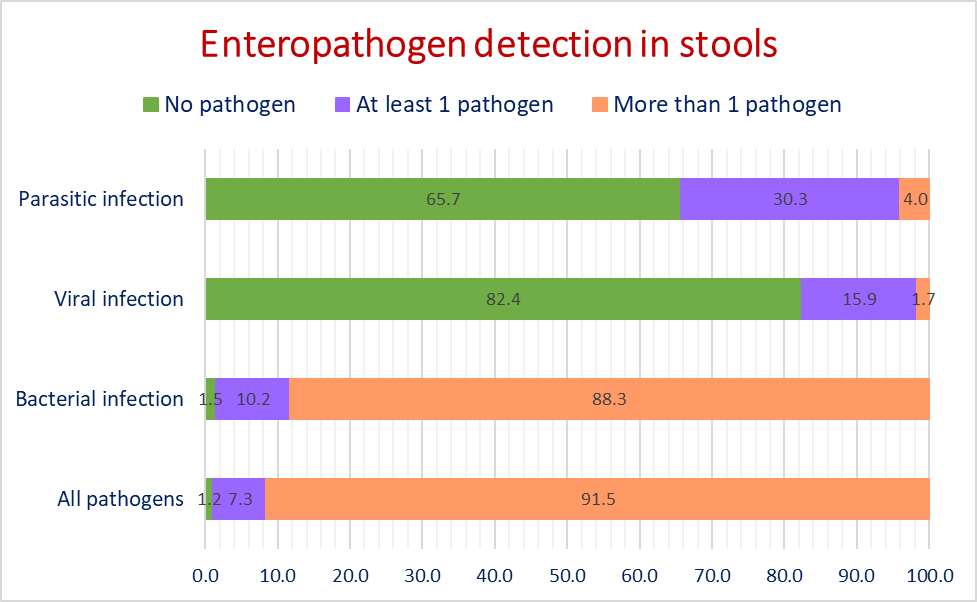


Fig D. Enteropathogen detection in the non-diarrheal stool samples collected from the study participants (n = 524). We have detected five types of intestinal parasites from the fecal samples of the study participants using TAC assays which are – Ascaris, Cryptosporidium, Giardia, Trichuris trichiura, and *Blastocystis* spp. In this figure, we did not consider infection with *Blastocystis* spp. as a parasitic infection because the pathogenic potential of Blastocystis spp. remains inconclusive.


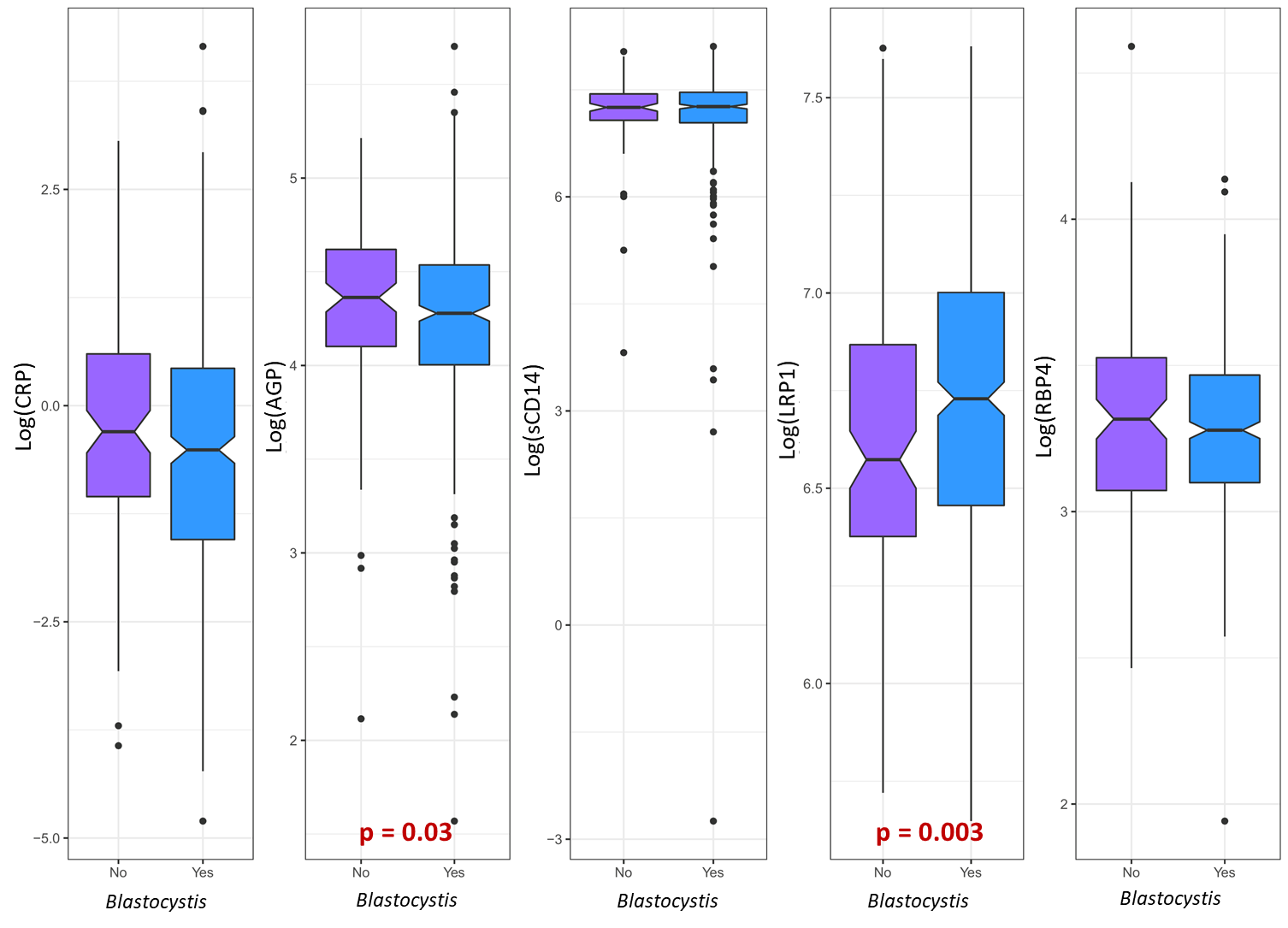


Fig E. Distribution of plasma biomarkers in adults (n = 524) with and without *Blastocystis*. CRP, C-reactive protein; AGP, alpha-1-acid glycoprotein; LRP1, low-density lipoprotein receptor-related protein-1; RBP4, retinol binding protein-4.


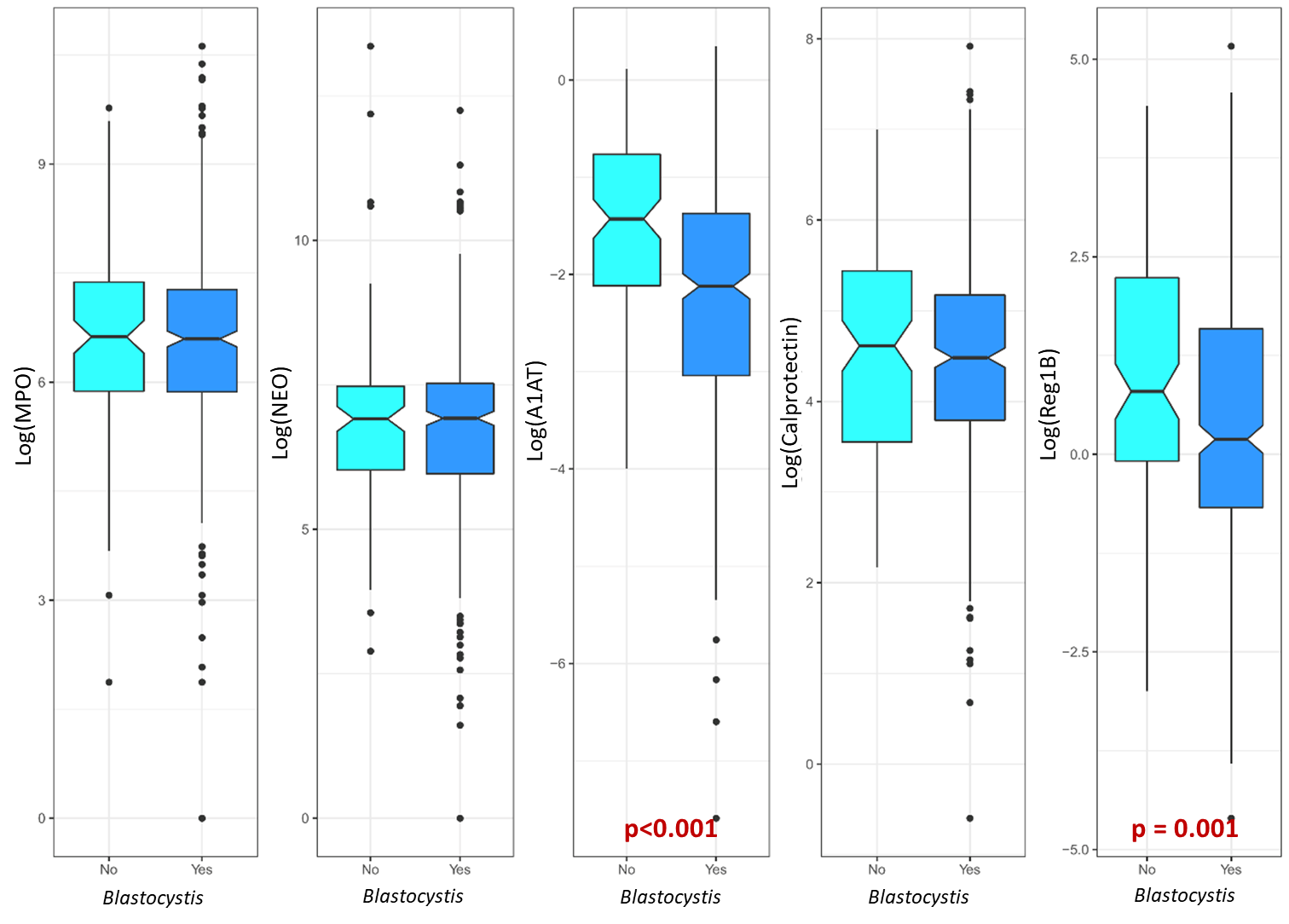


Fig F. Distribution of fecal biomarkers in adults (n = 524) with and without *Blastocystis*. MPO, myeloperoxidase; NEO, neopterin; A1AT, alpha-1 antitrypsin; Reg1B, Regenerating Family Member 1 Beta.


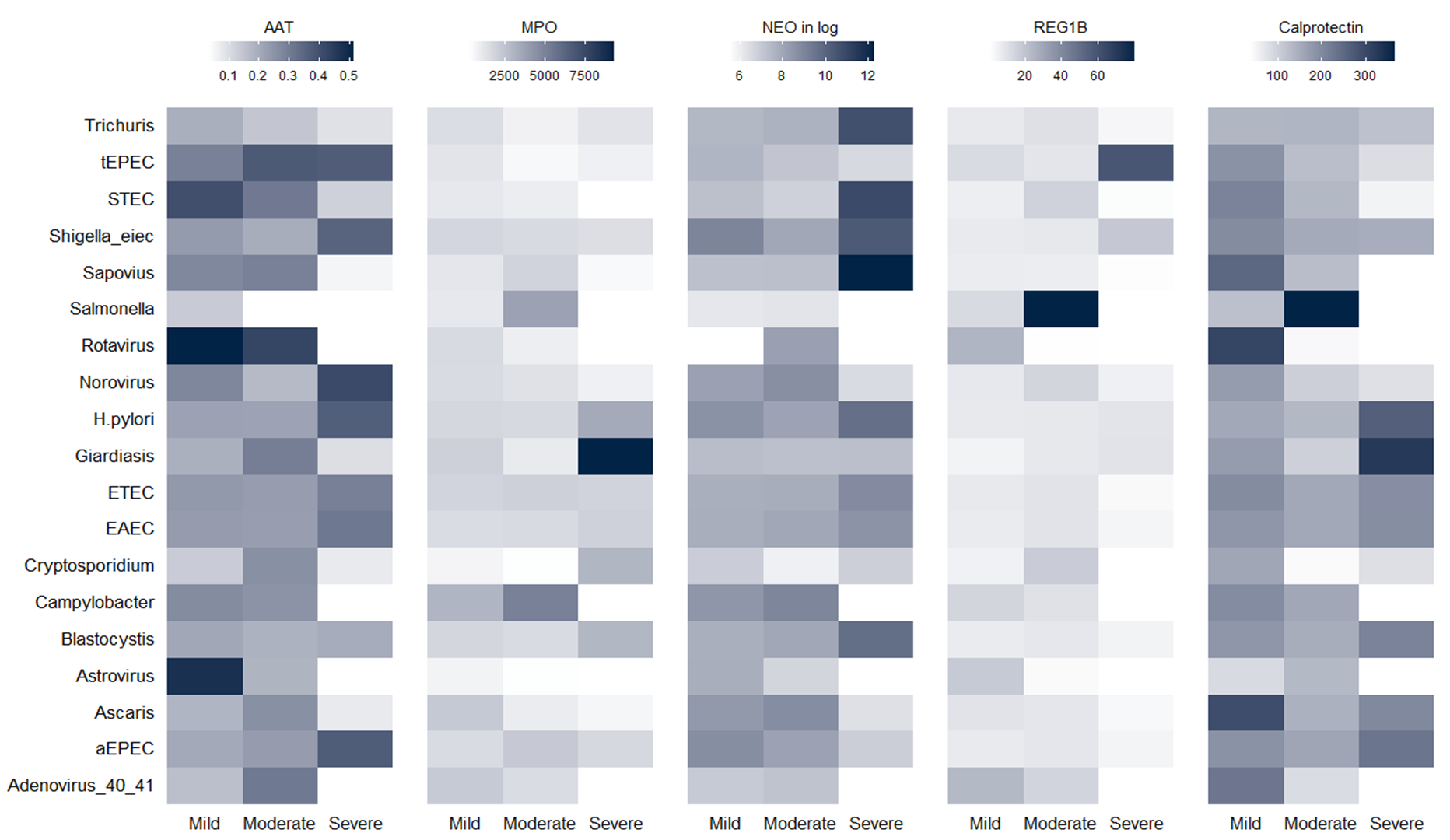


Fig G. Heatmap showing concentrations of fecal biomarkers of EED against positive results of enteric pathogens sorted by status of Body Mass Index (BMI) of the study participants. Herein, Mild, having a BMI between 17.0 to 18.4 kg/m^2^; Moderate, having a BMI between 16.0 to 16.9 kg/m^2^; Severe, having a BMI <16.0 kg/m^2^. AAT, alpha-1 antitrypsin; MPO, myeloperoxidase; NEO, neopterin; Reg1B, Regenerating Family Member 1 Beta.


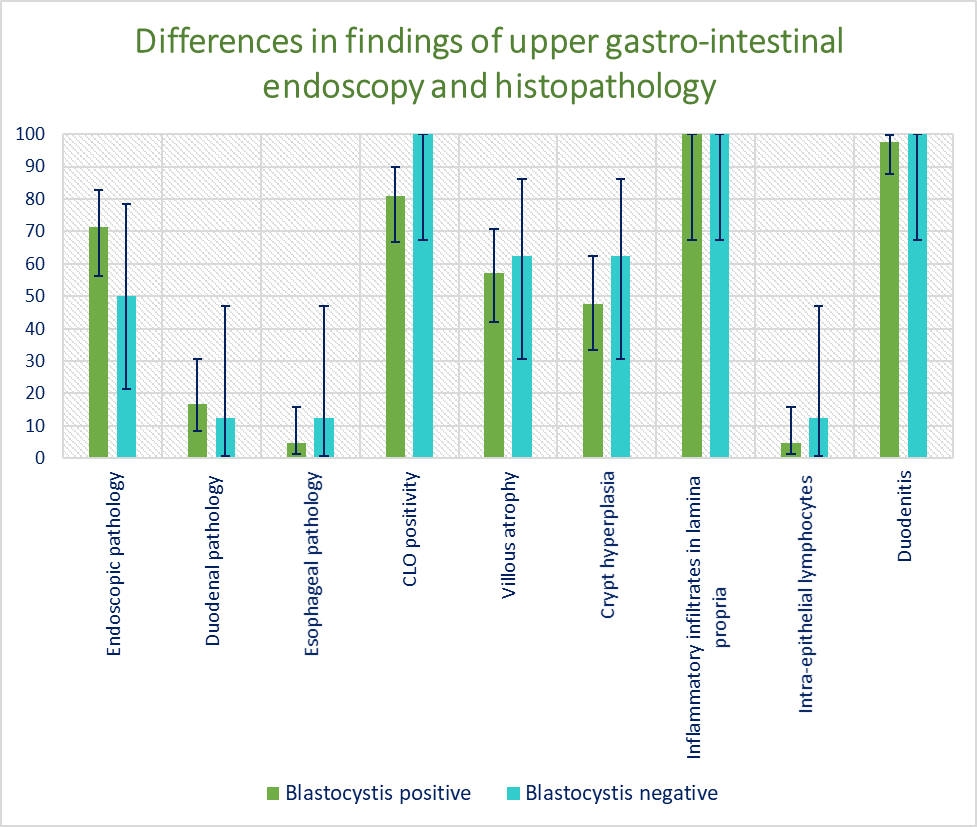
Fig H. Differences in findings of upper gastro-intestinal endoscopy and histopathology in participants tested positive and negative for *Blastocystis* spp. No significant differences were observed between *Blastocystis*-positive (n = 42) and *Blastocystis*-negative (n = 8) malnourished adults.
