# Supplementary material for: Infection with Blastocystis spp. and its association with enteric infections and environmental enteric dysfunction among slum-dwelling malnourished adults in Bangladesh
Source: PLoS Negl Trop Dis. 2021 Aug 18;15(8):e0009684. doi: 10.1371/journal.pntd.0009684 (PMC8405003; doi:10.1371/journal.pntd.0009684)
Supplement: S2 File — (DOCX) [file pntd.0009684.s003.docx]

**Table A. Name of investigating enteropathogens using TaqMan Array Card assay and their status of detection in this study**

| **Type of investigating pathogen** | **Name of investigating pathogen** | **Status of detection in this study** |
| --- | --- | --- |
| Virus | *Adenovirus 40/41* | Yes |
|  | *Astrovirus* | Yes |
|  | *Norovirus* | Yes |
|  | *Rotavirus* | Yes |
|  | *Sapovirus* | Yes |
| Bacteria | *Aeromonas* | Yes |
|  | *Bacteroides fragilis* | Yes |
|  | *Clostridium difficile* | Yes |
|  | *Campylobacter* | Yes |
|  | EAEC | Yes |
|  | aEPEC | Yes |
|  | tEPEC | Yes |
|  | ETEC | Yes |
|  | *Helicobacter pylori* | Yes |
|  | *M. tuberculosis* | Yes |
|  | *Plesiomonas* | Yes |
|  | *Salmonella* | Yes |
|  | *Shigella/EIEC* | Yes |
|  | STEC | Yes |
|  | *Vibrio cholerae* | Yes |
| Parasite | *Ancylostoma duodenale* | Yes |
|  | *Ascaris lumbricoides* | Yes |
|  | *Blastocystis spp.* | Yes |
|  | *Cryptosporidium* | Yes |
|  | *Cyclospora* | No |
|  | *Encephalitozoon intestinalis* | Yes |
|  | *Enterocytozoon bieneusi* | Yes |
|  | *Entamoeba histolytica* | Yes |
|  | *Giardia intestinalis* | Yes |
|  | *Hymenolepis nana* | Yes |
|  | *Isospora* | Yes |
|  | *Necator* | No |
|  | *Schistosoma* | No |
|  | *Strongyloides* | Yes |
|  | *Trichuris trichiura* | Yes |

**Table B. Association between *Blastocystis spp.* and fecal biomarkers of Environmental Enteric Dysfunction in malnourished adults using multivariable linear regression analyses^¶^**

| **Variables** | **MPO**, ng/mL | **NEO**, nmol/L | **Calprotectin**, μg/g | **AAT**, mg/g | **Reg1B**, μg/mL |
| --- | --- | --- | --- | --- | --- |
| Age (years) | -0.01 (-0.03, 0.004) | 0.01 (-0.01, 0.02) | -0.01 (-0.03, 0.002) | -0.03 (-0.04, -0.01)* | -0.02 (-0.05, 0.003) |
| Sex (Ref: female) |  |  |  |  |  |
| Male | -0.30 (-0.57, -0.04)* | -0.14 (-0.44, 0.16) | -0.20 (-0.44, 0.05) | 0.30 (0.001, 0.60)* | 0.32 (-0.07, 0.71) |
| Body Mass Index | 0.03 (-0.11, 0.16) | 0.01 ( -0.14, 0.16) | -0.01 (-0.13, 0.11) | 0.03 (-0.12, 0.19) | -0.09 (-0.29, 0.11) |
| Crowding Index (Ref: Low) |  |  |  |  |  |
| High |  |  |  | -0.32 (-0.64, 0.01) | -0.31 (-0.76, 0.13) |
| Season (Ref: Summer) |  |  |  |  |  |
| Monsson | 0.54 (0.18, 0.89)* | -0.69 (-1.09, -0.28)* | 0.15 (-0.17, 0.48) | 0.19 (-0.19, 0.56) | -0.24 (-0.77, 0.28) |
| Autumn | 0.40 (0.06, 0.74)* | -0.50 (-0.88, -0.13)* | 0.26 (-0.05, 0.56) | 0.05 (-0.32, 0.43) | 0.35 (-0.14, 0.84) |
| Winter | 0.49 (0.12, 0.87)* | -0.84 (-1.26, -0.42)* | -0.30 (-0.64, 0.04) | -0.01 (-0.39, 0.37) | 0.58 (0.03, 1.12)* |
| AGP |  | 0.006 (0.002, 0.01)* |  | 0.001 (-0.003, 0.004) | 0.002 (-0.002, 0.01) |
| Anemia |  |  |  | -0.15 (-0.41, 0.11) |  |
| aEPEC | 0.22 (-0.01, 0.44) |  |  | 0.02 (-0.23, 0.27) |  |
| *Trichuris trichiura* | -0.20 (-0.47, 0.07) |  | -0.20 (-0.44, 0.05) | -0.49 (-0.78, -0.20)* | -0.21 (-0.60, 0.18) |
| *Blastocystis spp.* | -0.04 (-0.31, 0.24) | -0.15 (-0.46, 0.16) | -0.05 (-0.30, 0.20) | -0.67 (-0.98, -0.36)* | -0.66 (-1.06, -0.26)* |

^¶^Each column represents an individual model. The results were obtained by multivariable linear regression analyses after adjusting for the variables with p-values <0.20 in the univariate linear regression analysis. All the fecal biomarkers were log-transformed prior to analysis. Herein, adjusted coefficient with 95% confidence interval (CI) has been reported. The asterisk (*) denotes the statistical significance with a p-value < 0.05. Abbreviations used: MPO, myeloperoxidase; NEO, neopterin; A1AT, alpha-1 anti-trypsin; Reg1B, Regenerating Family Member 1 Beta; AGP, alpha-1-acid glycoprotein; aEPEC, atypical strain of enteropathogenic *Escherichia coli*.

**Table C. Spearman’s correlations between CT-values for each pathogen detected by TaqMan Array Card assays and fecal biomarkers of Environmental Enteric Dysfunction^¶^**

| **Enteropathogens** | **MPO**, ng/mL | **NEO**, nmol/L | **Calprotectin**, μg/g | **AAT**, mg/g | **Reg1B**, μg/mL |
| --- | --- | --- | --- | --- | --- |
| Blastocystis spp. | 0.02 | -0.05 | 0.03 | 0.46* | 0.23* |
| *Cryptosporidium* | 0.05 | 0.08 | 0.06 | 0.02 | -0.03 |
| *Ascaris lumbricoides* | -0.01 | 0.02 | -0.07 | 0.02 | -0.05 |
| *Trichuris trichiura* | 0.05 | 0.07 | 0.07 | 0.18* | 0.10* |
| *Giardia intestinalis_a* | -0.01 | 0.02 | -0.03 | -0.03 | 0.05 |
| *Giardia intestinalis_b* | -0.03 | -0.03 | 0.01 | -0.03 | -0.001 |
|  |  |  |  |  |  |
| *Rotavirus* | -0.05 | 0.02 | 0.01 | -0.10* | -0.02 |
| *Norovirus_gi* | 0.02 | -0.03 | 0.08 | 0.07 | 0.07 |
| *Norovirus_gii* | -0.05 | -0.08 | 0.01 | -0.05 | -0.04 |
| *Adenovirus 40/41* | -0.04 | -0.004 | -0.01 | -0.01 | -0.05 |
| *Astrovirus* | 0.05 | 0.003 | 0.02 | -0.04 | -0.02 |
| *Sapovirus* | 0.08 | -0.04 | -0.01 | -0.03 | 0.01 |
|  |  |  |  |  |  |
| *Campylobacter* | -0.06 | -0.08 | -0.02 | -0.08 | -0.04 |
| EAEC | 0.01 | -0.003 | -0.09* | -0.03 | 0.04 |
| ETEC | -0.05 | 0.06 | -0.09* | -0.08 | 0.05 |
| tEPEC | 0.01 | 0.01 | -0.02 | -0.07 | -0.07 |
| aEPEC | -0.11 | -0.02 | -0.08 | -0.01 | -0.03 |
| STEC | 0.02 | 0.03 | -0.05 | -0.12* | -0.06 |
| *Shigella/EIEC* | -0.05 | -0.04 | -0.17* | -0.01 | 0.05 |
| *Salmonella* | -0.04 | 0.07 | -0.04 | 0.04 | -0.09* |

^¶^The results were obtained by Spearman’s correlation tests and reported as correlation coefficients (*rho*). The asterisk (*) denotes the statistical significance with a p-value < 0.05. Abbreviations used: MPO, myeloperoxidase; NEO, neopterin; A1AT, alpha-1 anti-trypsin; Reg1B, Regenerating Family Member 1 Beta; AGP, alpha-1-acid glycoprotein; EAEC, enteroaggregative *Escherichia coli*; ETEC, enterotoxigenic *Escherichia coli*; tEPEC, typical strain of enteropathogenic *Escherichia coli*; aEPEC, atypical strain of enteropathogenic *Escherichia coli*; STEC, shiga toxin-producing *Escherichia coli*; EIEC, enteroinvasive *Escherichia coli*.
