# Supplementary material for: Infection with Blastocystis spp. and its association with enteric infections and environmental enteric dysfunction among slum-dwelling malnourished adults in Bangladesh
Source: PLoS Negl Trop Dis. 2021 Aug 18;15(8):e0009684. doi: 10.1371/journal.pntd.0009684 (PMC8405003; doi:10.1371/journal.pntd.0009684)
Supplement: Attachment — Submitted filename: S2 Supporting Figures (1).doc [file pntd.0009684.s005.doc]

**Infection with *Blastocystis ~~hominis~~* and associated outcomes in relation to enteric infections and environmental enteric dysfunction among slum-dwelling malnourished adults in Bangladesh**

Shah Mohammad Fahim, Md. Amran Gazi, Md. Mehedi Hasan, Md. Ashraful Alam, Subhasish Das, Mustafa Mahfuz, M Masudur Rahman, Rashidul Haque, Shafiqul Alam Sarker, Tahmeed Ahmed

PLEASE CORRECT ALL “B.hominis” as “*Blastocystis*” including inside of the figures.

Supplementary figure 1. Prevalence of micronutrient deficiencies among the study participants (n = 524), overall and in participants with and without *Blastocystis~~. hominis~~*.

Supplementary figure 2. Seasonal trend in the prevalence of *Blastocystis~~. hominis~~*. among the study participants (n = 524). The prevalence of *Blastocystis~~. hominis~~*. was greater in winter compared to other seasons, although the difference was statistically insignificant.

Supplementary figure 3. Enteropathogen detection in the non-diarrheal stool samples collected from the study participants (n = 524).

Please check Supp.Fig.3 parasite infection bar. Because you mentioned that 78.6% malnourished adults were tested positive for *Blastocystis*. In the related bar this prevalance is about 30.3+4.0%. Please clarify, It cannot be understanded.

Supplementary figure 4. Distribution of plasma biomarkers in adults (n = 524) with and without *Blastocystis~~. hominis~~*.. CRP, C-reactive protein; AGP, alpha-1-acid glycoprotein; LRP1, low-density lipoprotein receptor-related protein-1; RBP4, retinol binding protein-4.

Supplementary figure 5. Distribution of fecal biomarkers in adults (n = 524) with and without *Blastocystis~~. hominis~~*.. MPO, myeloperoxidase; NEO, neopterin; A1AT, alpha-1 antitrypsin; Reg1B, Regenerating Family Member 1 Beta.

Supplementary figure 6. Differences in findings of upper gastro-intestinal endoscopy and histopathology in participants tested positive and negative for *Blastocystis~~. hominis~~*.. No significant differences were observed between *Blastocystis*-positive (n = 42) and *Blastocystis*-negative (n = 8) malnourished adults.
