# Supplementary material for: Infection with Blastocystis spp. and its association with enteric infections and environmental enteric dysfunction among slum-dwelling malnourished adults in Bangladesh
Source: PLoS Negl Trop Dis. 2021 Aug 18;15(8):e0009684. doi: 10.1371/journal.pntd.0009684 (PMC8405003; doi:10.1371/journal.pntd.0009684)
Supplement: Attachment — Submitted filename: Point-by-point responses.docx [file pntd.0009684.s006.docx]

**Responses to the review comments**

Dear Editor and Reviewers,

Thank you very much for reviewing the manuscript titled "Infection with *Blastocystis* spp. and its association with enteric infections and environmental enteric dysfunction among slum-dwelling malnourished adults in Bangladesh". Please find below the responses to the comments made by editors as well as reviewers and a description of the changes made in the manuscript.

**Editor's Responses to Questions:**

All 3 reviewers have indicated that the current study is of interest to the scientific community. The authors should take note of the concerns and suggestions of the reviewers, with particular attention to the comments of Reviewer #2, including the need for some form of validation for the high % of Blastocystis infection among the cohort examined. The reviewers have also indicated more details on the methods, such as the collection procedure (e.g. how many samples collected) should be provided. Please note that the term Blastocystis hominis is no longer used and the accepted terminology is Blastocystis spp. The lack of subtype information should be discussed as a limitation of the study.

**Response:** We would like to express our sincere thanks for reviewing the manuscript and providing insightful comments. We have addressed the issues raised by the reviewers and revised the draft accordingly. We have elaborated the methodology as per the suggestions of reviewers, and also changed the term *Blastocystis hominis* to *Blastocystis* spp*.* We have now mentioned in our draft that lack of subtype information is a limitation of this study. However, we could not verify the high % of *Blastocystis* infection among the study participants using another recommended laboratory method which has been suggested by the Reviewer#2. In this analysis, we used data from the Bangladesh Environmental Enteric Dysfunction (BEED) Study. In BEED study, we collected fecal samples only at enrollment to perform TaqMan Array Card (TAC) assays. The study design, duration, and budget of the BEED study did not allow us to collect additional fecal samples and do parasitological examinations using any other methods. We agree with you that it would be useful if we could validate the results against any recommended method. However, we have acknowledged in our manuscript that the absence of another recommended method (e.g. microscopic examination or PCR) to verify or confirm the positivity of stool samples is an important limitation of this study.

**Reviewer's Responses to Questions:**

*<b>Key Review Criteria Required for Acceptance?</b></br></br>*

*As you describe the new analyses required for acceptance, please consider the following:</br></br>*

*<b>Methods</b></br></br>*

*-Are the objectives of the study clearly articulated with a clear testable hypothesis stated?</br>*

*-Is the study design appropriate to address the stated objectives?</br>*

*-Is the population clearly described and appropriate for the hypothesis being tested?</br>*

*-Is the sample size sufficient to ensure adequate power to address the hypothesis being tested?</br>*

*-Were correct statistical analysis used to support conclusions?</br>*

*-Are there concerns about ethical or regulatory requirements being met?</br></br>*

**Reviewer #1:**

**Comment:** The study is acceptable for publication. Study design is appropriate for the aim. Authors mentioned limitations of this study. Lacking of the healthy control group and subtype analysis of Blastocystis are the main limitations for this study. Some suggestions were written for correction in the main text and supp2.

Yes, the sample size is sufficient to ensure adequate power to address the hypothesis being tested

Yes, correct statistical analysis was used to support conclusions

Yes, there concerns about ethical or regulatory requirements are being met

**Response:** Thank you very much for your very positive comments on study design, sample size, statistical analyses, and ethical requirements. We have already discussed the limitations regarding healthy control group and subtype analysis in our draft. We did also edit the main text and supporting files as per your suggestions.

**Reviewer #2:**

**Comment:** It is a routine practice in parasitological examinations to examine three faecal samples for each patient (such samples being taken at appropriate intervals). It is not specified how many samples per one patient were analyzed and how many times each trial was repeated. This information should be provided.

**Response:** We agree that examination of three fecal samples is the routine practice to identify intestinal parasites. It is because the examination of single specimen may enhance the risk of false negative results. However, such practice is mostly applicable for microscopic examinations. It is evident that intestinal parasites shed intermittently, and therefore, the conventional microscopic method may miss the detection of pathogen if the sample processing is not done properly. In our study, we have tested single sample per participant and assay was done once for each sample using the TaqMan Array Cards (TAC). The TAC assay is a single-plex real-time PCR analysis that can detect multiple targets even with a very small copy numbers (as low as 0.1 to 500 copies per 1 µl of reaction mixture). This method has an average sensitivity and specificity of 85% and 77%, respectively when compared with conventional methods such as microscopic examination, culture, and immunoassay. The average sensitivity and specificity of TAC analysis was recorded even higher (98% and 96%, respectively) when compared with laboratory-developed PCR-Luminex assays (1). Therefore, considering the analytical performance of TAC assay compared to other laboratory methods, we have analyzed single sample per participant and the assay was done once for each fecal sample. We have included this information in the methodology of the manuscript (page 7, line 130-139).

**Comment:** The percentage of infected patients is very high. Therefore, the results for positive samples should be confirmed/verified using another method recommended by CDC (e.g. fecal smears, PCR).

**Response:** Thank you for the comment. This analysis was cross-sectional in manner, and done as part of a much larger research programme on environmental enteric dysfunction. Therefore, we did collect fecal samples only at enrollment and perform TaqMan Array Card (TAC) assay which is a quantitative PCR method. We do agree with your comment that it would be better if we could collect multiple fecal samples to analyze, and verify the results by another recommended method. However, due to limitation of budget and time we could not collect additional samples and do parasitological examinations using any other methods. However, it is evident that TAC assay is a highly sensitive molecular diagnostic method for determination of a wide range of enteropathogens including intestinal parasites. We have discussed this in our manuscript (page 7, line 133-139 and page 18, line 341-343).

**Reviewer #3:**

Comment: Minor Revision. Partly related to STROBE Items 6, 9, and 13. While the inclusion and exclusion criteria is specified, the paper refers to the BEED study protocol in BMJ Open for details. The reference is a protocol that describes the recruitment process, but does not describe the outcome of recruitment. The paper can refer to a publication with the outcome of recruitment, or if this is the first from that protocol, perhaps provide more details in the Results section. Undernutrition in Bangladesh between females and males show a higher prevalence of malnutrition among females (23% vs 20%; https://linkprotect.cudasvc.com/url?a=https%3a%2f%2fglobalnutritionreport.org%2fresources%2fnutrition-profiles%2fasia%2fsouthern-asia%2fbangladesh%2f&c=E,1,gVlX4OzwpZp4e9_-b7oTo-wK0NDiUk3uclRqTB0nR7RLCgu-8z7-vzvnhwnCsYg2dmvASAd3Qh0htip6EmdZZdZLok5oGswkdRbBunL9AnxSz3aHhLoHE8LkHA,,&typo=1). However, the study has 73% females. What steps in the recruitment process resulted in this ratio?

**Response:** Thank you so much for your valuable comment. We have included a participant flowchart in the supporting files (S2: supplementary figure 1) that would probably answer your queries related to STROBE items 6, 9, and 13. We admit that almost three-fourth of our study participants were female. In this analysis, we have used data from the ongoing BEED study which is a community-based nutrition intervention trial. The study was conducted in a slum-setting where the number of migrating and floating people is substantial. This is to be noted that the rate of migration is higher in male population, particularly due to job opportunity and daily living. In this study, the participants were provided directly observed nutrition intervention for two months. Therefore, it was mandatory to visit the study site in every morning for two consecutive months in order to receive the nutritional therapy. Most of the male participants became ineligible as they informed that they may not stay in this slum setting for two months, and may migrate to another place. In addition, a substantial number of eligible males were workers or wage-earners, while most of the females were homemakers or part-time employees. Therefore, the refusal rate was higher among males as they were not agreed to miss the work for an hour to receive nutrition intervention. Hence, males are only one-fourth of our study participants. However, we have discussed this issue as a limitation of our study in the manuscript (page 18, line 330-337).

You have requested to provide the outcome of recruitment. The objectives of the BEED study include investigating the effectiveness of nutrition intervention in improving the nutritional status of the participants, examining the role of enteric pathogens in the pathogenesis of EED and malnutrition, and development of a histological scoring system to identify EED and validate the score against non-invasive biomarkers of EED. As the study is ongoing, it is not possible to report the outcome of the study. We have used only the baseline data for this analysis, and our goals were to determine the factors associated with *Blastocystis* spp. and investigate its association with fecal biomarkers of EED in malnourished adults living in a slum. Therefore, we have only reported the findings related to our objectives in this manuscript.

--------------------

*<b>Results</b></br></br>*

*-Does the analysis presented match the analysis plan?</br>*

*-Are the results clearly and completely presented?</br>*

*-Are the figures (Tables, Images) of sufficient quality for clarity?</br></br>*

**Reviewer #1:**

Yes, the analysis was presented match the analysis plan.

Yes, the results are clearly and completely presented

Yes, the figures (Tables, Images) of sufficient are quality for clarity

**Response:** Thank you very much for the positive comment.

**Reviewer #2:**

**Comment:** Molecular method used in these study -Quantitative PCR assay using the TaqMan Array Cards- allows obtaining quantitative data. Such data should be presented and correlated with the indications of the studied biomarkers. The ms needs to be supplemented with this.

**Response:** Thank you for the suggestion. We completely agree with you that quantitative PCR assay using TaqMan Array Cards (TAC) provides quantitative CT values for detection of enteropathogens. According to the published literature and manual of the cards that we have used, a CT value less than 35 is considered positive, while a CT value more than or equal to 35 is considered negative for the specific enteropathogen. In our case, the highest detectable CT value is 35. The software is set in such a way that it automatically considers all the values ≥35 as a constant value of “35”. The quality control check of our TAC assays was done meticulously, and the quality control team has also confirmed a CT value of 35 for all the negative samples (CT value≥35). Therefore, all the CT values for negative cases are constant and that is 35 in this analysis. In this regard, it may not be statistically valid to perform correlation between CT values and biomarkers, as we do not have the exact values for the negative samples. We are hopeful that you will understand the limitation and consider the issue.

**Comment:** Results with regard to association of Blastocystis hominis with fecal biomarkers should be summarized in a table.

**Response:** We have now summarized the results with regard to association of Blastocystis spp. with fecal biomarkers in a table in supplementary files (S3: supplementary table 2).

Reviewer #3: Minor Revision. Related to the comments in Methods. It might be useful to describe the flow of recruitment to consent and reasons for refusal to better understand the ratio of females to males in this study.

**Response:** We have included a flowchart in the supporting files (S2: supplementary figure 1) showing the flow of recruitment. We have also discussed the reasons for high refusal rate in males which results in higher number of female participants in the study (page 18, line 330-337).

--------------------

*<b>Conclusions</b></br></br>*

*-Are the conclusions supported by the data presented?</br>*

*-Are the limitations of analysis clearly described?</br>*

*-Do the authors discuss how these data can be helpful to advance our understanding of the topic under study?</br>*

*-Is public health relevance addressed?</br></br>*

**Reviewer #1:**

Yes, the conclusions are supported by the data presented

Yes, the limitations of analysis are clearly described

Yes, the authors discuss how these data can be helpful to advance our understanding of the topic under study.

**Response:** We would like to render our thanks for your very positive comment.

**Reviewer #2:**

**Comment:** The most common parasitic infections in Bangladesh are Ascariasis, Hookworm, Trichuriasis, and Cryptosporidium. However, in the ms it is not explained why only co-invasion with T. trichiura was taken into account.

**Response:** Thank you for pointing out the co-invasion of Blastocystis spp. with Ascariasis, Hookworm infestation, and Cryptosporidium in addition to Trichuriasis. In our study participants, the prevalence of Ascariasis, Hookworm infestation, and Cryptosporidiosis were 4%, 0%, and 2%. On the other hand, 26% of the participants were tested positive for *T. trichiura*. We also did not observe any significant relationship of *Blastocystis spp.* with Ascariasis, Hookworm infestation, and Cryptosporidiosis in regression analysis. Therefore, we only discussed on the co-invasion of *Blastocystis* spp. with *T. trichiura*. However, now we have explained the issue in discussion (page 17, line 307-313).

**Comment:** The high results of Blastocystis hominis vs. study participants and associated factors should be discussed.

**Response:** We have now discussed about the high results of Blastocystis hominis vs. study participants and associated factors in the manuscript.

**Reviewer #3:**

**Comment:** Minor revisions. Related to Strobe Item 19 and points above. Should the female: male ratio in this study be considered a limitation? How would this affect internal and external validity.

**Response:** We would like to express thanks to you for suggesting minor revisions to our manuscript. We admit that female: male ratio of this study is a limitation. We have discussed above about the causes of disproportion in recruitment of males and females in this study. We have also included it as a limitation in our manuscript. However, we have adjusted the regression models with sex of the participants in order to ascertain the internal and external validity of our results.

**Comment:** The title uses the phrase "associated outcomes" which tend to suggest to me, that the outcomes are a result of B. hominis infection. The study design is cross-sectional and given that is the case, any association of B. hominis infection and fecal biomarkers and other pathogens is just that - an association. I think the paper is best served by stating this as associations clearly.

**Response:** Thank you very much for your comment. We also agree with you and therefore, revised the title of the manuscript as per your suggestion.

--------------------

*<b>Editorial and Data Presentation Modifications?</b></br><br/>*

*Use this section for editorial suggestions as well as relatively minor modifications of existing data that would enhance clarity. If the only modifications needed are minor and/or editorial, you may wish to recommend “Minor Revision” or “Accept”.*

**Reviewer #1:** The only modifications needed are minor. So, my decision is minor revision. Authors should be made all of them.

**Response:** Thank you so much. We have revised the manuscript as per your suggestions.

**Reviewer #2:**

**Reviewer #3:**

**Comment:** Consider the best use of graphs and tables as some of the figures can easily be represented by a table. For instance, figure 1 shows the prevalence of B. hominis according to different sub-groups, but do not show the confidence intervals that would have demonstrated that the differences are not significant and reinforced the text. That information could be contained in a table or a graph of a point estimate bounded by 95% confidence intervals.

**Response:** Thank you for the suggestion. We have edited the figure 1 and included 95% confidence intervals.

**Comment:** Figure 2 shows the prevalence of infection of each pathogen defined by the TAC assay. This could also have been visualized as a table or heatmap which could also show the extent of co-infection. This information would also reinforce the text. I am particularly interested in the distribution of positive results of all enteropathogens and enteric biomarkers as a categorical or quantitative variable sorted by BMI. By choosing to show aggregate data, you lose the value of the powerful methods you have used. Hence the suggestion of a heatmap above. I think this might in fact be very useful to explore the how infection / inflammation (whether due to B hominis or not) is distributed across the range of malnourished adults, if only to generate new hypotheses.

**Response:** Thank you for your suggestion. We have prepared a heatmaps as per your suggestion and included it in the supporting files (supplementary figure 5). The figure shows that concentrations of A1AT, MPO, NEO, and Calprotectin were elevated in *Blastocystis*-positive malnourished adults who were severely underweight (BMI<16.0 kg/m^2^). This figure is also useful in understanding the distribution of fecal biomarkers of EED in relation to infection with different enteropathogens across the ranges of BMI in malnourished adults. We have also described the figure in result section of the manuscript (page 12, line 222-228).

Lines 204-207 and 227-230 may be repeating information on REG1B. If it is, perhaps, just place that information in one place.

**Response:** The results are different. The first one is distribution of REG1B concentrations measured in the stool samples of adults with and without Blastocystis spp., while the second one is the result of multivariable linear regression analysis that demonstrates the association of *Blastocystis* with fecal REG1B concentrations.

--------------------

*<b>Summary and General Comments</b></br></br>*

*Use this section to provide overall comments, discuss strengths/weaknesses of the study, novelty, significance, general execution and scholarship. You may also include additional comments for the author, including concerns about dual publication, research ethics, or publication ethics. If requesting major revision, please articulate the new experiments that are needed.*

**Reviewer #1:**

**Comment:** I think the publication of this study, which is aimed at explaining the pathogenesis of the Blastocystis protist parasite and includes a large number of samples, will make a great contribution to the researcher. It will benefit both in terms of creating new hypotheses and in terms of evaluating the researchers ' own work with the data of this study. However, as the authors noted, the absence of a particularly healthy control group and the absence of subtype analysis of Blastocystis, as well as the absence of microscopic examination of stool samples, are an important limitation. It will be useful for the TaqMan array method used to indicate the pathogens it is investigating and present these identified pathogens in a table. A standard investigation of all enteric pathogens and the study of a large number of patients and a detailed survey are the strengths of the study.

**Response:** Thank you very much for your comment. We have now included the strengths and limitations of our study as per your suggestion. We have also prepared a table (S3: supplementary table 1) to present the pathogens that has been investigated and detected by the TaqMan Array Card assays.

**Reviewer #2:**

**Comment:** This ms presents the data on Blastocystis prevalence in malnourished adult patients in Bangladesh. The authors investigated the relationship of B. hominis with the occurrence of intestinal biomarkers. The data are interesting and can be used to develop new diagnostic methods. However, I have some doubts about the parasitological research. Analysis of results is clear but the parasitological analysis is insufficient. They should be completed.

**Response:** Thank you very much for your positive comment regarding our analysis and findings. We also agree with you regarding insufficient parasitological analysis. It would definitely be interesting if we could use another recommended method to verify the positivity of *Blastocystis spp*. However, we used data from the Bangladesh Environmental Enteric Dysfunction (BEED) Study for this analysis. In BEED study, we collected fecal samples only at enrollment to perform TaqMan Array Card (TAC) assays. The study design, duration, and budget of the BEED study did not allow us to collect additional fecal samples and do parasitological examinations using any other methods. We agree with you that it would be useful if we could validate the results against any recommended method. However, we have discussed the issue in the limitation of our manuscript.

**Reviewer #3:**

**Comment:** This is a relevant paper and addresses the possible associations of B. hominis infection with other pathogens and host factors.

**Response:** Thank you for the positive comment.

--------------------

*PLOS authors have the option to publish the peer review history of their article (<a href="https://linkprotect.cudasvc.com/url?a=https%3a%2f%2fjournals.plos.org%2fplosntds%2fs%2feditorial-and-peer-review-process%23loc-peer-review-history&c=E,1,6Z5G1gviFVm3_xysdgMwXR662gpKL7zNJuaoC9tODlMB564GkYNNoOvTwp4YhW5CpAolqG_cq1Jn4EjhvaIQkBOc6eHIHFz0fUbSHBEPqtfFxoWGWui7CB5Xu9rD&typo=1" target="_blank">what does this mean?</a>). If published, this will include your full peer review and any attached files.<br><br>*

*If you choose “no”, your identity will remain anonymous but your review may still be made public.<br><br>*

*<b>Do you want your identity to be public for this peer review?</b> For information about this choice, including consent withdrawal, please see our <a href="https://linkprotect.cudasvc.com/url?a=https%3a%2f%2fwww.plos.org%2fprivacy-policy&c=E,1,nst4gqVTXRHE33WluSl4g8fZtOmm1I0xj9qWt0w89OdRISoBOJyVNJkGwzZWVEeceGsVAF1Kv6Juo0wxiFf8QNH2cW6kAqutIdopcP-bPu47HFZV&typo=1" target="_blank">Privacy Policy</a>.*

Reviewer #1: No

Reviewer #2: No

Reviewer #3: No

**Response:** Thank you for reviewing our draft and providing insightful comments.

**Comments on Main Text**

**Abstract**

**Comment:** Please remove “hominis”. Only *Blastocystis* or *Blastocystis* sp. could be used. All *Blastocystis* in the whole text should be written italic.

**Response:** Thank you for the comment. We have changed it accordingly.

**Comment:** *Blastocystis* is a protist. Please change this.

**Response:** We have changed it accordingly.

**Results**

**Comment:** Please add results as % and p value.

**Response:** We have included % and p value in the results where you have mentioned to add (page 10, line 178-183).

**Comment:** Please check line 180-181 and clarify.

**Response:** Thank you for the comment. Line 180-181 describes the differences in prevalence of *Blastocystis* in different seasons. We performed chi-square test to determine the statistical significance, and found a p-value of 0.06. However, line 212-213 describes the results of multivariable logistic regression analysis where we included “Season” as an explanatory variable, and it showed significant association with *Blastocystis* infection in the multivariable model.

**Comment:** You can write (significant p-value) bold

**Response:** Thank you. We have written the significant p-values in bold letters in Table 2.

**Comment:** That is mean the differences is statistically significant. However, the p-value is 0.09. Which of them is important for comparison?

**Response:** The first p-value is the result of bi-variate analysis, and the second one is of multivariable analysis. The second one is important as that is the adjusted p-value.

**Comments on S2 Supporting Figures**

Supplementary figure 3: Please check Supp.Fig.3 parasite infection bar. Because you mentioned that 78.6% malnourished adults were tested positive for Blastocystis. In the related bar this prevalence is about 30.3+4.0%. Please clarify, it cannot be understood.

**Response:** We have detected five types of intestinal parasites from the fecal samples of the study participants using TAC assays which are – Ascaris, Cryptosporidium, Giardia, Trichuris trichiura, and *Blastocystis* spp. In this figure, we did not consider infection with *Blastocystis* spp. as parasitic infection because the pathogenic potential of *Blastocystis* spp. remains inconclusive. Hence, the prevalence of parasitic infection is reported 30.3%+4.0% considering the infection with Ascaris, Cryptosporidium, Giardia, and *Trichuris trichiura*. We have now included this information in the figure legend.

**References:**

1.	Liu J, Gratz J, Amour C, Kibiki G, Becker S, Janaki L, et al. A laboratory-developed TaqMan Array Card for simultaneous detection of 19 enteropathogens. Journal of clinical microbiology. 2013;51(2):472-80.
