# Supplementary material for: Infection with Blastocystis spp. and its association with enteric infections and environmental enteric dysfunction among slum-dwelling malnourished adults in Bangladesh
Source: PLoS Negl Trop Dis. 2021 Aug 18;15(8):e0009684. doi: 10.1371/journal.pntd.0009684 (PMC8405003; doi:10.1371/journal.pntd.0009684)
Supplement: Attachment — Submitted filename: Point-by-point responses.docx [file pntd.0009684.s007.docx]

**Responses to the review comments**

Dear Editor and Reviewers,

Thank you very much for reviewing the manuscript titled "Infection with *Blastocystis* spp. and its association with enteric infections and environmental enteric dysfunction among slum-dwelling malnourished adults in Bangladesh". Please find below the responses to the comments made by editors as well as reviewers and a description of the changes made in the manuscript.

**Editor's Responses to Questions:**

Thank you very much for submitting your manuscript "Infection with Blastocystis spp. and its association with enteric infections and environmental enteric dysfunction among slum-dwelling malnourished adults in Bangladesh" for consideration at PLOS Neglected Tropical Diseases. As with all papers reviewed by the journal, your manuscript was reviewed by members of the editorial board and by several independent reviewers. The reviewers appreciated the attention to an important topic. Based on the reviews, we are likely to accept this manuscript for publication, providing that you modify the manuscript according to the review recommendations.

Response: Thank you very much for reviewing the manuscript and providing very positive comments regarding the manuscript. We did modify the draft according to the review recommendations.

The reviewers have completed their assessment of the revised manuscript. Reviewers #1 and #3 are supportive of accepting the current version. However, Reviewer #2 has indicated 2 minor edits to the manuscript before acceptance. These are to provide details and correlations of the TaqMan qPCR data with the assessed biomarkers, and to provide details on lab identification of Ascariasis, Hookworm infestation, Entamoeba histolytica, and Cryptosporidium species, which is currently missing in the manuscript. Both these aspects can be included in the supplementary section of the manuscript.

Response: Many thanks for your assessment and insightful remarks. We have revised our draft accordingly. We have included a table showing the correlations between TaqMan qPCR data and fecal biomarkers in the supplementary section (S3: Supplementary table 3). In this study, we did identify all the enteropathogens including ascaris lumbricoides, hookworm, *Entamoeba histolytica*, and *Cryptosporidium* species using the TaqMan Array Cards (TAC) assays. This information is mentioned in the methods (page 6, line 127-128). We have also included a table in the supplementary section (S3: Supplementary table 1) showing the list of pathogens detected in this study using TAC assays.

**Reviewer's Responses to Questions:**

<b>Key Review Criteria Required for Acceptance?</b></br></br>

As you describe the new analyses required for acceptance, please consider the following:</br></br>

<b>Methods</b></br></br>

-Are the objectives of the study clearly articulated with a clear testable hypothesis stated?</br>

-Is the study design appropriate to address the stated objectives?</br>

-Is the population clearly described and appropriate for the hypothesis being tested?</br>

-Is the sample size sufficient to ensure adequate power to address the hypothesis being tested?</br>

-Were correct statistical analysis used to support conclusions?</br>

-Are there concerns about ethical or regulatory requirements being met?</br></br>

Reviewer #1: Yes

Response: Thank you.

Reviewer #2: It is a routine practice in parasitological examinations to examine three fecal samples for each patient (such samples being taken at appropriate intervals). The present research has only been done from one trial. The authors suggest that the method used in this study has a high detection rate. However, they do not take into account the fact that dispersive forms of parasites can be excreted periodically and taking only one sample for analysis significantly reduces the detection of, for example, parasite eggs.

Response: Thank you for the comment. We completely agree with you that it would be useful if we could examine three fecal samples for each patient. However, the study design, duration, and budget did not allow us to collect additional fecal samples and do repeated parasitological examinations. We have acknowledged the issue as an important limitation of this study in our manuscript (page 18, line 344-346).

Molecular method used in the study -Quantitative PCR assay using the TaqMan Array Cards - allows obtaining quantitative data. Such data should be presented and correlated with the indications of the studied biomarkers. The manuscript needs to be supplemented with this.

Response: We did perform correlations between quantitative PCR data and biomarkersWe have included a table showing the correlations between TaqMan qPCR data and fecal biomarkers in the supplementary section (S3: Supplementary table 3).

Reviewer #3: (No Response)

Response: Thank you.

--------------------

<b>Results</b></br></br>

-Does the analysis presented match the analysis plan?</br>

-Are the results clearly and completely presented?</br>

-Are the figures (Tables, Images) of sufficient quality for clarity?</br></br>

Reviewer #1: Yes

Response: Thank you.

Reviewer #2: (No Response)

Response: Thank you.

Reviewer #3: (No Response)

Response: Thank you.

--------------------

<b>Conclusions</b></br></br>

-Are the conclusions supported by the data presented?</br>

-Are the limitations of analysis clearly described?</br>

-Do the authors discuss how these data can be helpful to advance our understanding of the topic under study?</br>

-Is public health relevance addressed?</br></br>

Reviewer #1: Yes

Response: Thank you.

Reviewer #2: The discussion in lines 311-314 ["However, the prevalence of Ascariasis, Hookworm infestation, Entamoeba histolytica, and Cryptosporidium species were inconsiderable (4%, 0.8%, 1%, and 2%, respectively) among the study participants which may explain the insignificant results of co-invasion between Blastocystis and these parasites."] refers to results that are not described in the relevant sections of the paper, i.e.: materials and methods and the results. It is not known how these species of parasites were detected and why these studies were not described in Methods and Results.

Response: Thank you for your comment. In this study, we did identify all the enteropathogens including ascaris lumbricoides, hookworm, *Entamoeba histolytica*, and *Cryptosporidium* species using the TaqMan Array Cards (TAC) assays. This information is mentioned in the methods (page 6, line 127-128). We have also included a table in the supplementary section (S3: Supplementary table 1) showing the list of pathogens detected in this study using TAC assays.

Reviewer #3: (No Response)

Response: Thank you.

--------------------

<b>Editorial and Data Presentation Modifications?</b></br><br/>

Use this section for editorial suggestions as well as relatively minor modifications of existing data that would enhance clarity. If the only modifications needed are minor and/or editorial, you may wish to recommend “Minor Revision” or “Accept”.

Reviewer #1: Revised manuscript Line 311 "Ascariasis, Hookworm infestation" should be changed as "ascariasis, hookworm infection".

After his correction was made there is no need reviewing again by me.

Response: We have made the changes as per your suggestion. Thank you.

Reviewer #2: (No Response)

Response: Thank you.

Reviewer #3: (No Response)

Response: Thank you.

--------------------

<b>Summary and General Comments</b></br></br>

Use this section to provide overall comments, discuss strengths/weaknesses of the study, novelty, significance, general execution and scholarship. You may also include additional comments for the author, including concerns about dual publication, research ethics, or publication ethics. If requesting major revision, please articulate the new experiments that are needed.

Reviewer #1: Dear Editor

Thank you very much for reviewing invitation again for this manuscript.

I checked all my suggestion and other reviewer’s comments. Authors tried to make all of corrections according to the reviewer's comments.

In this revised form, the manuscript has become better understood and scientifically enriched.

My decision is "accept" of this manuscript for publication.

Kind regards

Response: Thank you so much for your very positive comments.

Reviewer #2: This manuscript presents the data on Blastocystis prevalence in malnourished adult patients in Bangladesh. The authors investigated the relationship of B. hominis with the occurrence of intestinal biomarkers. The data are interesting and can be used to develop new diagnostic methods. However, I have some doubts about the parasitological research.

The analysis of the results is clear but the parasitological analysis is still insufficient.

Response: Thank you so much for your constructive remarks. We have addressed your comments and revised our draft accordingly. We have also discussed the limitations of this study elaborately in our manuscript.

Reviewer #3: (No Response)

Response: Thank you.

--------------------

PLOS authors have the option to publish the peer review history of their article (<a href="https://linkprotect.cudasvc.com/url?a=https%3a%2f%2fjournals.plos.org%2fplosntds%2fs%2feditorial-and-peer-review-process%23loc-peer-review-history&c=E,1,SOV9Z0KtIXrYOtdjwslRJvMnFHZoGUS_gmRgkftK4ArFxMgCk8ZJ6JeVZKbyUiNP23C5vnZ2ugb41Flgl6jx5s5PZY_7KzJ5j0sMch_2&typo=1" target="_blank">what does this mean?</a>). If published, this will include your full peer review and any attached files.<br><br>

If you choose “no”, your identity will remain anonymous but your review may still be made public.<br><br>

<b>Do you want your identity to be public for this peer review?</b> For information about this choice, including consent withdrawal, please see our <a href="https://linkprotect.cudasvc.com/url?a=https%3a%2f%2fwww.plos.org%2fprivacy-policy&c=E,1,a4FKmLRAYk9qYoc9v9v23nqFD0f4M3AP95mC8C6n6PCjyujbPfLPe--_kGrgJOu9ullU2-v9jbVawJWE0_lUxqte-bA5zdlaferbr92few,,&typo=1" target="_blank">Privacy Policy</a>.

Reviewer #1: No

Reviewer #2: No

Reviewer #3: No

Response: Thank you for reviewing the manuscript.
